# Medical resource inventory model for emergency preparation with uncertain demand and stochastic occurrence time under considering different risk preferences at the airport

**DOI:** 10.1371/journal.pone.0183472

**Published:** 2017-09-20

**Authors:** Wei Pan, Ying Guo, Lei Jin, ShuJie Liao

**Affiliations:** 1 School of Economic and Management, Wuhan University, Wuhan, China; 2 School of Management, Shandong University, Jinan, China; 3 Cancer Biology Research Center, Tongji Hospital, Tongji Medical College, Huazhong University of Science and Technology, Wuhan, Hubei, PR China; University of Florida, UNITED STATES

## Abstract

With the high accident rate of civil aviation, medical resource inventory becomes more important for emergency management at the airport. Meanwhile, medical products usually are time-sensitive and short lifetime. Moreover, we find that the optimal medical resource inventory depends on multiple factors such as different risk preferences, the material shelf life and so on. Thus, it becomes very complex in a real-life environment. According to this situation, we construct medical resource inventory decision model for emergency preparation at the airport. Our model is formulated in such a way as to simultaneously consider uncertain demand, stochastic occurrence time and different risk preferences. For solving this problem, a new programming is developed. Finally, a numerical example is presented to illustrate the proposed method. The results show that it is effective for determining the optimal medical resource inventory for emergency preparation with uncertain demand and stochastic occurrence time under considering different risk preferences at the airport.

## Introduction

In recent years, many organizations and countries are confronted with the dilemma between the lack of efficient emergency management and the increase in death. In the event of an aviation accident, there are very high likelihood of human injury and death. Therefore, our research is motivated by the disaster relief which deals with rescuing casualties when a civil aviation disaster has occurred. In such environment, scholars and managers are more concerned about airport emergency management than before.

In fact, an intrinsic feature of emergency preparation system is the demand is uncertain, which may lead to large amount of shortage costs. Furthermore, according to the unpredictable occurrence time about a civil aviation disaster with the time-sensitive medical resource, we find that the out-of-date phenomenon of medical inventory is common. In addition, risk attitude is related to the severity of disaster and occurrence probability in emergency and violate situations. Therefore, we study an emergency inventory management system under considering uncertain demand, stochastic occurrence time, limited life-time of medical resource and different risk attitudes for minimizing the cost about shortage and expiration.

We solve the best level of medical inventory for emergency under considering criteria of risk neutral and risk aversion with the newsboy model. In our model, we find that uncertain demand plays a significant role in the inventory system and should not be overlooked because shortages and surplus have attributed to emergency financial issues. Furthermore, stochastic occurrence time is also considered in this paper. Medical resource supply for emergency at the airport should be kept in a certain inventory level, which is in conformity with the reality that occurrence time is random. We study the optimal medical resource inventory level for minimizing the cost in emergency preparation. Optimal inventory levels will not only satisfy the basic requirement of the government and people but also reduce the waste of resources. We believe operation of the airport and the social emergency rescue will more efficient than before.

In this paper, we analyze the following questions: How should be medical resource inventory level under considering uncertaindemand and random occurrence time? How can different risk attitudes, uncertain demand and stochastic occurrence time affect medical resource inventory level? Finally, what are the results under considering different risk attitudes?

The rest of this paper is organized as follows. Section 2 presents the emergency management literature about s inventory. Section 3 describes the problem and gives the research assumptions. Section 4 develops the stochastic model under considering criteria of risk neutral, risk-averse and risk-taking. Meanwhile, we give the analytic solutions to the problem. In section 5, we discuss the results. Numerical examples are also presented in this section in order to give some interpretations. In section 6, we assume the risk-averse degree is a function of the probability of a disaster during shelf life for extending this research. The last section is the conclusion and further ideas.

## Literature review

With the development of emergency management, more and more scholars and researchers have paid attention to inventory problem about disaster relief. Beamon and Kotleba (2006) constructed the inventory model of complex and emergency situation referring the global and established inventory control model with stochastic demand. Then, this model was applied to the rescue practice in South Sudan Civil War and they found that the emergency inventory model could benefit a lot for emergency rescue [1]. Whybark (2007) researched inventory management of the disaster relief. Meanwhile, they specifically pointed out the acquisition, storage, and distribution of emergency supplies. These problems are very important for emergency for reducing the losses of life and property in disaster [2]. Under considering the significant function of emergency inventory, many scholars and researchers respectively studied inventory site selection, inventory scope, distribution of emergency supplies and so on (Selda & Emmett, 2010; Serhan et al., 2011; Rawls & Turnquist, 2010; Mete & Zabinsky, 2010) [3–6]. Kunz et al (2014) provided a new methodology for reducing disaster consequences. Meanwhile, it implied the increasing investment about emergency abilities (e.g. employee training, early communication with the customs in disaster-prone areas and so on) [7]. Huseyin & Zelda (2010) studied the stochastic optimization problem about medical supplies in emergency preparedness. The stochastic optimization problem mainly considered the inventory levels and distribution decisions in a single emergency management for one accident, including preparation and response phase [6]. Rubel & Shinya (2014) established an emergency inventory model with stochastic demand and lead time under assuming the uniform distribution of demand and lead time. Furthermore, they considered that uniformed distribution was common and reasonable for analyzing the stochastic variables in the emergency and violate situations [8].

Logistics and distribution are also very important for emergency resource management. Holguín-Veras (2007) [9] described the key logistical issues that plagued the response to disasters, making out that the design of reliable emergency logistics was hampered by lack of knowledge, methods, and scientific analysis. Their research was studied on the basis of Hurricane Katrina event and field interview methods. Wassenhove (2006) [10] pinpointed the cross learning potential for both the humanitarian and private sectors in emergency relief operations as well as possibilities of getting involved through corporate social responsibility. In addition, the author outlined strategies for better preparedness and the need for supply chains and stated the case for closer collaboration between humanitarians, businesses and academics to achieve better and more effective supply chains, in order to respond to the complexities of emergency logistics and relieving the lives of those blighted by disaster. Ozguven and Ozbay (2014) [11] did a literature review for various aspects of emergency inventory management for disasters. This article pointed out the characteristics of storage and delivery options for emergency supplies, classified the comprehensive inventory-related literature, and finally discussed the critical issues and key findings related to the emergency inventory management field, including suggestions for future research directions.

When attempting to manage emergency situations, the notion of risk attitude is an important consideration for decision-makers that must be measured (Noyan, 2012; Demirag, 2013) [12–13]. In order to deal with this problem, the CVaR (conditional value at risk) methodology has proven increasingly popular since Rockafellar and Uryasev (2000; 2002) [14–15] first demonstrated its fundamental properties. In this regard, Wu et al., (2013) [16] studied the effect of uncertain capacity on the inventory decision on the VaR and CVaR methodologies. Their results showed that uncertain capacity affected the order quantity under the different risk-averse criterions. While much research has studied the classical newsboy problem with the CVaR method (Jammernegg and Kischka, 2007; 2009; 2013) [17–19], more steps should be taken to make sense of the combination effects of inventory management and risk attitudes (Piantadosi, 2008; Chan et al., 2014) [20–21]. Therefore, this paper differs from past studies in that the inventory management system for emergency materials with short life cycle by CVaR method is uncertain demand and stochastic occurrence time.

In the face of complex and changeable emergency situation, most of the literature only considered the uncertainty of demand or lead time. However, few literates studied random occurrence time of a disaster or occurrence rate during a fixed period. Moreover, the above literature neglected the perishable emergency supplies. Emergency supplies are mostly some perishable products, such as alcohol, sterile gauze, blood, special drugs and so on, all of which are time-sensitive. Optimizing the inventory level both reduces inventory cost for airport and saves social resources. In view of the short life cycle of perishable products, we used this method because the newsboy model is used for order and inventory decisions of products with short life cycle [22]. In this paper, the goal of the model is to achieve the minimum cost under the constraint of service quality level. At the same time, we take the stochastic occurrence time (or occurrence rate) of a disaster into consideration. Moreover, we compare the decisions between stochastic occurrence time and deterministic occurrence time.

Our paper differs from previous papers because it includes the following three features: 1) We assumed demand and occurrence time as random variables and considered the medical supplies as perishable emergency resources; 2) We analyzed the effects of random variables under considering different risk attitudes, including risk neutral, risk-averse and risk-taking; 3) When the risk-averse degree is a function of disaster probability, we observed how the probability affect the results.

## Assumption and model

Like other airport in the world, airports in china have reached the minimum standards on the emergency preparation required by the International Civil Aviation Organization (ICAO). Consisting with the contents of the rules to emergency rescue for civil transport airport and the equipment of emergency rescue for civil aviation transport airport, airports always be required to store a certain amount of medical supplies and equipment to prepare the preliminary relief. Furthermore, airports have a higher occurrence probability during the whole journey, and thus, it is necessary to reserve emergency resources for an airport. Due to the high-consequences and the high requirements of the public, airports tend to store more medical supplies and equipment. Since the demand of medical emergency supplies is uncertain and the emergency materials is time-sensitive, the financial issue may be the most critical problem because of shortage or oversupply, which will induce damage losses or waste from out-of-date.

The airport’s emergency medical supplies replenishment strategy is different from regular or quantitative replenishment strategy. At the end of one accident, airports continue to order medical supplies to prepare for next accident satisfying the demand of the initial aid as fully as possible. Obviously, they adopt the similar kind of (*S-1*, *S*) replenishment strategy, but they make *S* as big as possible. In this way, airports will take up a certain amount of current capital, and the losses caused by expired cost are also very high, leading airports’ high investment of emergency preparedness. Under the constraint of service quality level, to achieve the minimizing costs is one of the problems considered by each airport. Optimizing the inventory level both reduces inventory cost for airport and saves social resources.

As a result of the low-probability, it is reasonable to hypothesize that the probability of two or more accidents happened at the same time even between a short interval is zero. In addition, it should be pointed out that the emergency preparatory stage is a period from the time of one accident after rescue to the next accident happened. From above assumption, the ending time of a single accident emergency preparation will be conveyed as *min (t*, *T)*, where *T* is shelf-life of emergency medical supplies and *t* is the occurrence time of a disaster.

In this paper, emergency inventory levels *I* in a single phase is the decision variables. For single phase, we can use the classical newsboy model for modeling analysis, and the classical newsboy model mainly researches order and inventory decisions with stochastic demand for short life products. Different from the classical newsboy model, we take occurrence time *t* and demand *x* are random variables. The corresponding density function and cumulative distribution function is *f*(*x*),*g*(*t*);*F*(*x*),*G*(*t*) respectively. The two random distributions are independent. *SL* is the predefined service quality level.

**Fig 1:**
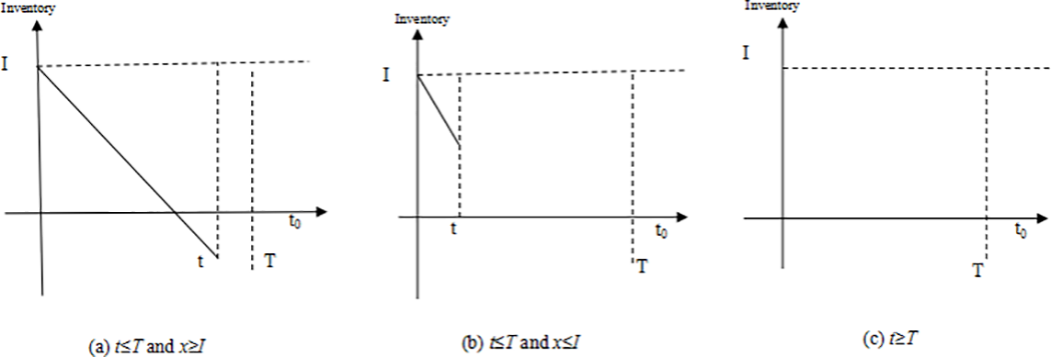
Scenarios during a single stage of emergency preparedness.

Fig 1 shows a random occurrence time and stochastic demand in a single preparedness. In case (a), there are out of stock and shortage losses; In the case of (c), all materials expire and there are overdue losses; In case (b), some emergency supplies have salvages at the end of this emergency preparedness’ stage. Similar to single phase newsboy model of surplus value, we assumed *θ* residual quantity will be lost, with 0 ≤ *θ* ≤ 1.

## Models and solutions

Facing with the low-probability and high-consequence disaster, decision-makers may behave different for different risk attitudes, including risk neutral, risk-taking and risk-averse. Some may be risk-taking for the low-probability, and some other may be risk-adverse for high-consequence. In order to validate the relationship between effect degree and risk aversion, we also considered the criteria of risk neutral at the same time. The goal of this article optimizes costs under satisfying the constraint of a certain service quality level. Costs include expired cost and shortage loss. *e* is the unit cost for out of date and *s* is unit cost for out of stock, assuming *e < s*.

### Risk neutral newsboy problem

1) Because of the low-probability and high-consequences, we define the service level from the unmet view. The service level is the probability when the unmet demand must be below the unacceptable level 1-*SL*, then:

$$p(x\geq I)=\int_{0}^{T}\int_{I}^{+\infty }f(x)g(t)dxdt\leq 1-SL$$ (1)

2) Expected loss

(a) *t* ≤ *T* and *x* ≥ *I*

$$C_{a}=\int_{I}^{+\infty }s(x-I)f(x)dx$$ (2)

(b) *t* ≤ *T* and *x* ≤ *I*

$$C_{b}=\int_{0}^{I}\theta e(I-x)f(x)dx$$ (3)

(c)*t* ≥ *T*

$$C_{c}=\int_{0}^{+\infty }eIf(x)dx$$ (4)

$$\begin{array}{l} ETC=E_{a}+E_{b}+E_{c}\\ =\int_{0}^{T}\int_{I}^{+\infty }s(x-I)f(x)g(t)dxdt+\int_{0}^{T}\int_{0}^{I}\theta e(I-x)f(x)g(t)dxdt+\int_{T}^{+\infty }\int_{0}^{+\infty }eIf(x)g(t)dxdt \end{array}$$ (5)

$$dETC/dI=-\int_{0}^{T}\int_{I}^{+\infty }sf(x)g(t)dxdt+\int_{0}^{T}\int_{0}^{I}\theta ef(x)g(t)dxdt+\int_{T}^{+\infty }\int_{0}^{+\infty }ef(x)g(t)dxdt$$ (6)

$$d^{2}ETC/dI^{2}=sf(I)G(T)+\theta ef(I)G(T)>0$$ (7)

According to the sign of the second derivative (Eq (7)), we judged that the total expected loss is a convex function of inventory *I*. So there is the only optimal solution for the optimal value. Let the first derivative (Eq (6)) equate to zero, then:

−s(1−F(I))G(T)+θeF(I)G(T)+e(1−G(T))=0 (8)

$$\mathrm{And}\;F(I^{*})=\frac{(s+e)G(T)-e}{\left(s+\theta e)G(T\right)},I^{*}=F^{-1}(\min(1,{\left(\frac{(s+e)G(T)-e}{\left(s+\theta e)G(T\right)}\right)}^{+}))$$ (9)

From service level objective meeting the formula (1), we can be obtained:

$$I\geq F^{-1}({\left(1-\frac{1-SL}{G(T)}\right)}^{+})$$ (10)

If Eq (9) meets non-Eq (10), we have:

$I^{*}=F^{-1}(\min(1,{\left(\frac{(s+e)G(T)-e}{\left(s+\theta e)G(T\right)}\right)}^{+}))$, or else, we conclude: $I=F^{-1}({\left(1-\frac{1-SL}{G(T)}\right)}^{+})$.

When $I=F^{-1}({\left(1-\frac{1-SL}{G(T)}\right)}^{+})$, I* increases with increasing of shelf life *T* and the service level *SL*.

When $I^{*}=F^{-1}(\min(1,{\left(\frac{(s+e)G(T)-e}{\left(s+\theta e)G(T\right)}\right)}^{+}))$, I* increases with increasing of shelf life *T* and the unit cost for out of stock *s*. Meanwhile, it decreases with increasing of unit cost for out of date *e* and loss ratio *θ*. (Proof. S1 Appendix)

From above equations, we can conclude that under the criteria of risk neutral, the optimal inventory level is not only related to shelf life, but also related to occurrence time and random distributions of stochastic variables.

### Risk-averse newsboy problem

Considering risk factors, we use the tool of conditional value at risk (CVaR) in the field of financial risk management [23], assuming that the degree of risk aversion is *η*, referring to the literature research results, we have:

$$CVaR(TC(I))=\underset{v\in R}{\min}\{L(I,v)≔v+\frac{1}{1-\eta }E{\left[TC(I)-v\right]}^{+}\}$$ (11)

To minimize the upper bound of losses, we set objective function as minimizing the CVaR:

$$\underset{I}{\min}\;CVaR(TC(I))=\underset{I}{\min}\;\underset{v\in R}{\min}\{L(I,v)≔v+\frac{1}{1-\eta }E{\left[TC(I)-v\right]}^{+}\}$$

$$\begin{array}{l} L(I,v)=v+\frac{1}{1-\eta }\int_{0}^{T}\int_{I}^{+\infty }{\left(s(x-I)-v\right)}^{+}f(x)g(t)dxdt\\ \;+\frac{1}{1-\eta }\int_{0}^{T}\int_{0}^{I}{\left(\theta e(I-x)-v\right)}^{+}f(x)g(t)dxdt+\frac{1}{1-\eta }\int_{T}^{+\infty }\int_{0}^{+\infty }{\left(eI-v\right)}^{+}f(x)g(t)dxdt \end{array}$$ (12)

Calculating the optimal value of Eq (12), we can obtain the optimal solution of decision variable *I*: (Proof. S1 Appendix)

*i:* When $\frac{(s+e)G(T)-e}{s}<\eta$, $I^{*}=F^{-1}({\left(1-\frac{1-\eta }{G(T)}\right)}^{+})\frac{s}{s+e}$.

*ii:* When $\frac{(s+e)G(T)-e}{s}>\eta$, $I^{*}=F^{-1}(\min(1,\frac{\eta }{G(T)}))\frac{s}{s+\theta e}$.

*iii:* When $\frac{(s+e)G(T)-e}{s}=\eta$ and *G*(*T*) ≥ *η*, then I* can be any value within the available range $I^{*}\in (F^{-1}(\min(1,\frac{\eta }{G(T)}))\frac{s}{s+e},F^{-1}(\min(1,\frac{\eta }{G(T)}))\frac{s}{s+\theta e}]$.

The constraint of service quality level is an expected value and do not include random variables, so there is no risk in constraint and we cannot use the tool of CVaR to measure the risk. Then we obtain the non-equation from formula (1): $I\geq F^{-1}({\left(1-\frac{1-SL}{G(T)}\right)}^{+})$, and the optimal inventory level I*should meet the constraint of service quality level.

When $I=F^{-1}({\left(1-\frac{1-SL}{G(T)}\right)}^{+})$, I* increases with increasing of shelf life *T* and the service level *SL*.

When $I^{*}=F^{-1}({\left(1-\frac{1-\eta }{G(T)}\right)}^{+})\frac{s}{s+e}$, I* increases with increasing of shelf life *T* and the unit cost for out of stock *s*. Meanwhile, it decreases with increasing of unit fee from out-of-date *e*, without relating with the loss ratio *θ*.

When $I^{*}=F^{-1}(\min(1,\frac{\eta }{G(T)}))\frac{s}{s+\theta e}$, I* decreases with increasing of shelf life *T* and the unit cost for out of stock *s*. Meanwhile, it decreases with increasing of unit overdue fee *e* and loss ratio *θ*.

From above equations, we can conclude that under the criteria of risk-averse, the optimal inventory level is related to not only shelf life but also occurrence time and random distributions of stochastic variables.

### Risk-taking newsboy problem

CVaR only focus on the loss exceeded VaR, which present a pessimism. However, facing with low-probability events, some decision-makers behave optimistically to prepare disasters [24]. As a risk-taking decision-maker, he/she always focus on the fraction less-than VaR. We note the conditional value at risk with risk-taking CVaRT. The definition of CVaRT can be described as the following formulation.

CVaRT(TC(I))=E(TC(I)|TC(I)≤VaR)

Based on literature researches, we can solve the CVaRT by the following formulation, and *α* is the degree of risk-taking.

$$CVaRT(TC(I))={\left(1-\alpha \right)}^{-1}[E(TC(I))-\alpha CVaR_{1-\alpha }(TC(I))]$$

The objective of our model is to minimize CVaRT*(TC(I))*. We calculate CVaR_1-α_(*TC(I)*) first, then seek the optimal inventory level I* to minimize CVaRT*(TC(I))*.

*i:* When $\frac{\left(s+e)(1-G(T)\right)}{s}>\alpha$, $I^{*}=F^{-1}(\min(1,{\left(\frac{(s+e)G(T)-s\alpha -e}{\left(s+\theta e)G(T\right)}\right)}^{+}))$.

*ii:* When $\frac{\left(s+e)(1-G(T)\right)}{s}\leq \alpha$, $I^{*}=F^{-1}(\min(1,\frac{s(1-\alpha )}{\left(s+\theta e)G(T\right)}))$.

The constraint of service quality level is an expected value and do not include random variables, there is no risk in constraint and we cannot use the tool of CVaRT to measure the risk.

When $I=F^{-1}({\left(1-\frac{1-SL}{G(T)}\right)}^{+})$, I* increases with increasing of shelf life *T* and increases with increasing of the service level *SL*.

When $I^{*}=F^{-1}(\min(1,{\left(\frac{(s+e)G(T)-s\alpha -e}{\left(s+\theta e)G(T\right)}\right)}^{+}))$, I* increases with increasing of shelf life *T* and the unit cost for out of stock *s*. Meanwhile, it decreases with increasing of unit overdue fee *e* and the loss ratio *θ*.

When $I^{*}=F^{-1}(\min(1,\frac{s(1-\alpha )}{\left(s+\theta e)G(T\right)}))$, I* decreases with increasing of shelf life *T* and the unit cost for out of stock *s*. Meanwhile, it decreases with increasing of unit overdue fee *e* and loss ratio *θ*.

From above equations, we can conclude that under the criteria of risk-taking, the optimal inventory level is related to not only shelf life but also occurrence time and random distributions of stochastic variables.

## Results and discussions

In classical newsboy model [16], the replenishment period is fixed *T*, and the optimal decision under criteria of risk neutral is $\bar{I}=F^{-1}(\frac{s}{s+e})$, and the optimal decision under risk-averse criteria is $\bar{I}=F^{-1}(\frac{(1-\eta )s}{s+e})$. The optimal decision under risk-taking criteria is $\bar{I}=F^{-1}(\frac{\alpha s}{s+e})$. It is should be pointed that we assume the optimal solution in our model meet the constraint of service level in the following discussion. The assumption is reasonable when discussing a low-probability and high-consequences disaster. It is available to compare our model to classical newsboy model, because they are all single period with random demand. The aim is just to measure the effects of random occurrence time on decision with different risk attitudes.

### Result analysis

First, we analyze effects of random occurrence time on the risk neutral, risk-averse and risk-taking decision-making. In addition to comparing the analytical solutions, this article also has carried on analysis of numerical example to explain the results.

Conclusion 1: under the rule of risk neutral, the longer shelf life is, the larger the optimal inventory I* is. The following two reasons may illuminate it. One is that the replenishment period *min* (*t*, *T*) may become longer, leading the prepared inventory lager. The other one is that the larger optimal inventory I* can avoid shortage and make the probability of expire lower, just because the probability of a disaster is higher during *T*.

Conclusion 2: under the risk-averse rule, when η is greater, that is, when $\frac{(s+e)G(T)-e}{s}<\eta$, the optimal solution is $I^{*}=F^{-1}({\left(1-\frac{1-\eta }{G(T)}\right)}^{+})\frac{s}{s+e}$. I* increases with increasing of shelf life *T*. The most possible interpretation is that when the degree of risk aversion *η* is larger, the expected loss will be greater during longer shelf life time because of the randomness of occurrence time, and they will keep high inventory to balance the shortage risk. It will be opposite when *η* is smaller. When *η* is small, $\frac{(s+e)G(T)-e}{s}\geq \eta$. $I^{*}=F^{-1}(\min(1,\frac{\eta }{G(T)}))\frac{s}{s+\theta e}$, I* decreases with increasing of shelf life *T*. As a result, there is a turning point for the degree of risk aversion. When less than the turning point, I* decreases with increment of shelf life *T*, and I* increases with increasing of shelf life *T* when the degree of risk aversion is greater than the turning point.

Conclusion 3: under the risk-taking rule, when *α* is smaller, $\frac{\left(s+e)(1-G(T)\right)}{s}>\alpha$, the optimal solution is $I^{*}=F^{-1}(\min(1,{\left(\frac{(s+e)G(T)-s\alpha -e}{\left(s+\theta e)G(T\right)}\right)}^{+}))$. I* increases with increasing of shelf life *T*. The most possible interpretation is that when the risk-taking degree *α* is smaller, the expected loss will be greater during longer shelf life time because of the randomness of occurrence time, and they will keep high inventory to balance the shortage risk. It will be opposite when *α* is larger. When *α* is larger, $\frac{\left(s+e)(1-G(T)\right)}{s}\leq \alpha$, $I^{*}=F^{-1}(\min(1,{\left(\frac{s(1-\alpha )}{\left(s+\theta e)G(T\right)}\right)}^{+}))$. I* decreases with increasing of shelf life *T*. As a result, there is a turning point for the risk-taking degree. When less than the turning point, I* increases with increment of shelf life *T*, and I* decreases with increasing of shelf life *T* when the risk-taking degree is greater than the turning point.

Conclusion 4: under the rule of risk neutral, $\bar{I}>I^{*}$, $I^{*}=F^{-1}(\min(1,{\left(\frac{(s+e)G(T)-e}{\left(s+\theta e)G(T\right)}\right)}^{+}))$, that is, the randomness of occurrence time leading the lower optimal inventory level. This shows that the inventory of emergency resources is affected by stochastic occurrence time. Theoretically, if the occurrence time is uncertain, the holding level with stochastic occurrence time should be same with deterministic occurrence time under the rule of risk neutral. However, we assume that the end time of a single emergency preparedness is *min (t*, *T)*, so the interval time between orders is shortened reducing the quantities of orders.

Conclusion 5: under the risk-averse rule, when $G(T)\leq \frac{s+e}{2s+e}$ and $\frac{(s+e)G(T)-e}{s}<\eta$, then there is ${\hat{\eta }}_{1}\in (1-G(T),1)$. We have $\bar{I}\geq I^{*}$ when $\eta \leq {\hat{\eta }}_{1}$, and we obtain $\bar{I}\leq I^{*}$ when $\eta \geq {\hat{\eta }}_{1}$. When $G(T)\geq \frac{s+e}{2s+e}$ and $\frac{(s+e)G(T)-e}{s}<\eta$, then there is ${\hat{\eta }}_{1}\in (\frac{(s+e)G(T)-e}{s},1)$. We have $\bar{I}\geq I^{*}$ when $\eta \leq {\hat{\eta }}_{1}$, and we obtain $\bar{I}\leq I^{*}$ when $\eta \geq {\hat{\eta }}_{1}$. When $\eta <\frac{(s+e)G(T)-e}{s}$, we always obtain $\bar{I}\geq I^{*}$. So there is a turning point for *η*, when *η* is bigger than the turning point, the optimal inventory level under stochastic occurrence time is higher than that under deterministic occurrence time. The result is opposite when *η* is smaller than the turning point. (Proof. S1 Appendix)

Conclusion 6: under the risk-taking rule, there is *G*_1_(*T*) ∈ (0,1), *G*_2_(*T*) ∈ (0,1), $\alpha_{1}\in (0,\frac{\left(s+e)(1-G(T)\right)}{s})$ and $\alpha_{2}\in (\frac{\left(s+e)(1-G(T)\right)}{s},1)$. When *G*(*T*) ≥ *G*_1_(*T*), we always have $\bar{I}\leq I^{*}$ within $0\leq \alpha \leq \frac{\left(s+e)(1-G(T)\right)}{s}$. When *G*(*T*) ≤ *G*_1_(*T*), 0 ≤ *α* ≤ *α*_1_, we have $\bar{I}\leq I^{*}$. When *G*(*T*) ≤ *G*_1_(*T*), $\alpha_{1}\leq \alpha \leq \frac{\left(s+e)(1-G(T)\right)}{s}$, we have $\bar{I}\geq I^{*}$. When *G*(*T*) ≤ *G*_2_(*T*), we always have $\bar{I}\geq I^{*}$ within $\frac{\left(s+e)(1-G(T)\right)}{s}\leq \alpha \leq 1$. When *G*(*T*) ≥ *G*_2_(*T*), *α*_2_ ≤ *α* ≤ 1, we have $\bar{I}\geq I^{*}$. When *G*(*T*) ≥ *G*_2_(*T*), $\frac{\left(s+e)(1-G(T)\right)}{s}\leq \alpha \leq \alpha_{2}$, we have $\bar{I}\leq I^{*}$. Obviously, the decision is affected by risk-taking degree and random distribution of occurrence time. If a decision-maker’s risk-taking degree is high and *G(T)* is low, which means he/she is more risk-taking and a disaster is less likely to happen, the decision result will be more bold. (Proof. S1 Appendix)

### Numerical example

We assume the parameters *s* = 3, *e* = 2, *θ* = 0.5. The random distribution of demand is uniform distribution during [2, 4], and based on the safety reports from ICAO, we assume the random distribution of occurrence time is uniform distribution during [1, 3].

**Fig 2:**
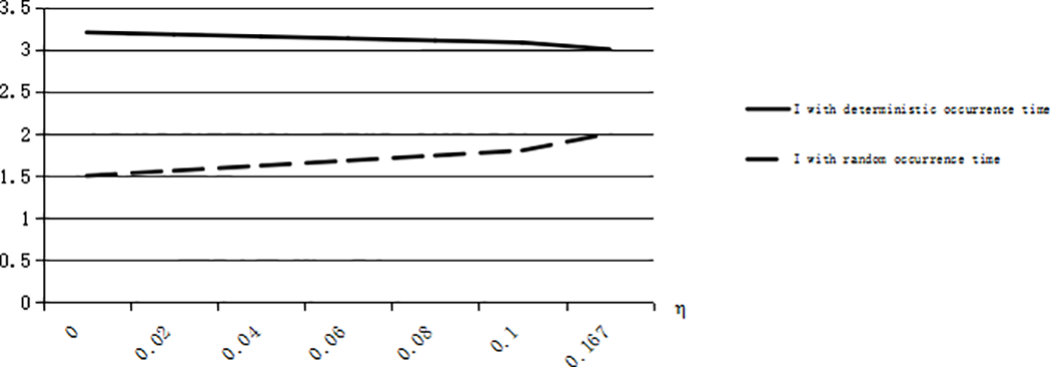
The risk-averse optimal inventory levels when G(T) = 0.5.

**Fig 3:**
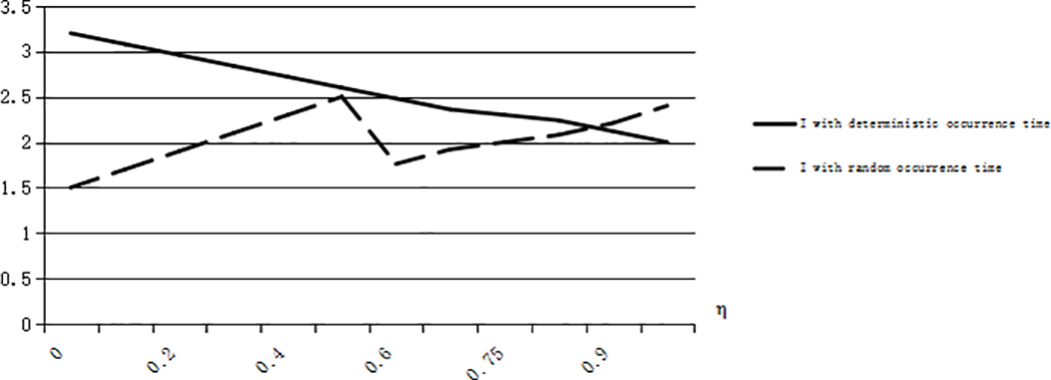
The risk-averse optimal inventory levels when G(T) = 0.75.

We can obtain that *G(T)* = 0.5 when *T* = 2, and ${\hat{\eta }}_{1}=8/9$. We calculate $\bar{I}=2.06,I^{*}=2.28$ when $\eta =0.95\geq {\hat{\eta }}_{1}$, and $\bar{I}=2.48,I^{*}=1.44$ when $1/6\leq \eta =0.6\leq {\hat{\eta }}_{1}$. From Fig 2, we can validate the conclusion 5 that $\bar{I}\geq I^{*}$ when $\eta <\frac{(s+e)G(T)-e}{s}$. From Fig 3, we can validate the conclusion 5 that there is ${\hat{\eta }}_{1}\in (\frac{(s+e)G(T)-e}{s},1)$ when $G(T)\geq \frac{s+e}{2s+e}$, meeting $\bar{I}\geq I^{*}$ when $\frac{(s+e)G(T)-e}{s}<\eta \leq {\hat{\eta }}_{1}$ and $\bar{I}\leq I^{*}$ when $\eta \geq {\hat{\eta }}_{1}$.

**Fig 4:**
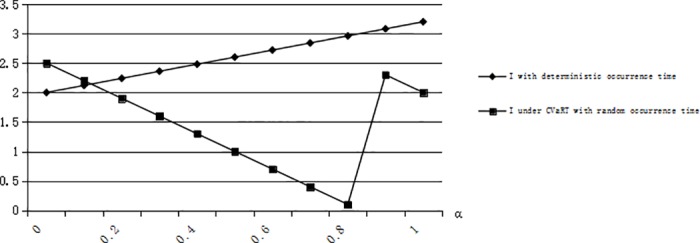
The risk-taking optimal inventory levels when G(T) = 0.5.

**Fig 5:**
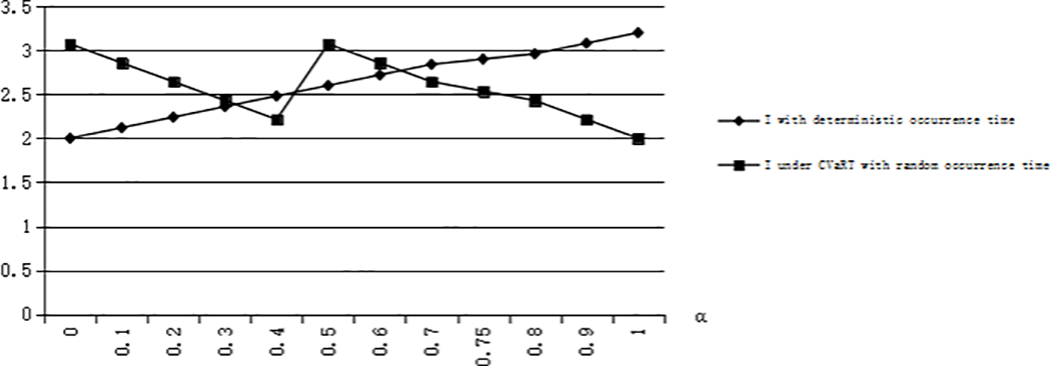
The risk-taking optimal inventory levels when G(T) = 0.7.

**Fig 6:**
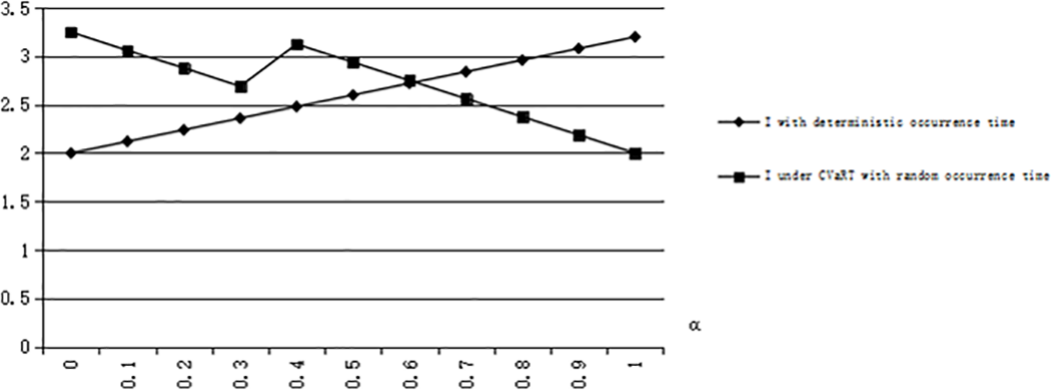
The risk-taking optimal inventory levels when G(T) = 0.8.

Let us illuminate conclusion 6 from Fig 4 to Fig 6. We can calculate that *G*_*1*_*(T)* = 0.77, *G*_*2*_*(T)* = 0.59. When *G(T)* = 0.5, $\frac{\left(s+e)(1-G(T)\right)}{s}=0.83$, *α*_1_ = 0.21, we have $\bar{I}\leq I^{*}$ if 0 ≤ *α* ≤ *α*_1_, and we have $\bar{I}\geq I^{*}$ if *α*_1_ ≤ *α* ≤ 1 just for *G*(*T*) ≤ *G*_2_(*T*) ≤ *G*_1_(*T*) (see Fig 4). When *G(T)* = 0.7, $\frac{\left(s+e)(1-G(T)\right)}{s}=0.5$, *α*_1_ = 0.32, *α*_2_ = 0.64, we have $\bar{I}\geq I^{*}$ if *α*_1_ ≤ *α* ≤ 0.5, *α*_2_ ≤ *α* ≤ 1 and we have $\bar{I}\leq I^{*}$ in other situations (see Fig 5). When *G(T)* = 0.8, $\frac{\left(s+e)(1-G(T)\right)}{s}=0.33$, *α*_2_ = 0.65, we have $\bar{I}\geq I^{*}$ if *α*_2_ ≤ *α* ≤ 1 and $\bar{I}\leq I^{*}$ in other situations (see Fig 6).

**Fig 7:**
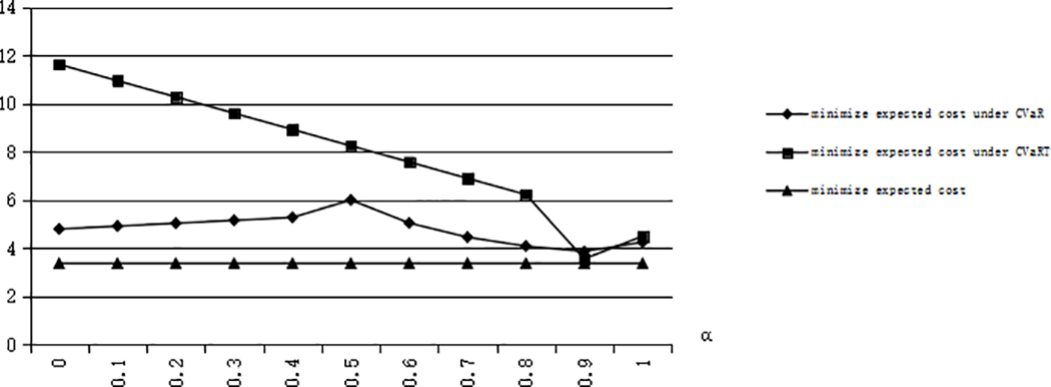
Optimal expected cost when G(T) = 0.5 with different risk attitudes.

From the constraint of service quality level, we have *CSL* = 1−(1−*F*(*I*))*G*(*T*), *CSL* is the cycle service level. So if the optimal inventory level *I* is higher, the cycle service level is higher. From Fig 7, we can see that the expected cost in expected theory has the lowest expected cost, the expected cost with CVaR has a higher cost than expected theory and the expected cost with CVaRT has the highest expected cost. The expected theory does not consider any risk just focus on expected cost. Risk-averse and risk-taking decision-makers obtain a higher expected cost considering uncertainty. If a decision-maker is risk-averse, he/she will decide to store more to decrease risk but increase the cost of out of date. If a decision-maker is risk-taking, he/she will store less than risk-neutral but increase the cost of shortage. Note that the unit shortage cost is larger than the unit cost of out of date. We also conclude that the with respect to the optimal inventory level of emergency resources, a risk-averse decision-maker does not always Pareto dominate a risk-taking decision-maker. Because the optimal inventory level for a risk-averse decision-maker does not always be higher for risk-taking decision-maker if a specific value of expected cost is the optimal value for a risk-averse and risk-taking decision-maker. The expected costs under risk-averse and risk-taking criteria are non-monotonous for *α*, and the reason is the changeable value of *G(T)* and the existence of turning point for *η* or α.

In conclusion, the random occurrence time affects the decision under both risk-averse and risk-taking criteria. In addition, the decisions with different risk attitude are made to seek balance between cost and risk. Under the risk-averse criterion, we first calculate the probability that the loss exceeds the maximum expected risk losses (VaR) [25]. Then we compare the value of the probability between models with stochastic occurrence time and deterministic occurrence time. If the former is lesser, the decision result shows greater degree of risk aversion, without realizing the Pareto optimality under the corresponding degree of risk aversion. So, in order to realize the Pareto optimality, they need to reduce inventory levels ($\bar{I}\geq I^{*}$), reducing the maximum expected risk losses but increasing the responsible risk. If the former is larger, the responsible risk with the decision is greater than the corresponding risk for the decision maker’s degree of risk aversion. So, in order to reduce risk, they need to increase inventory ($\bar{I}\leq I^{*}$), increasing the maximum expected losses. The determination of inventory level is the result of balance between risks and losses. Similarly, under the risk-taking criterion, if the probability of loss less than VaR with occurrence time is larger than that with deterministic occurrence time, the responsible risk with the decision is lower than the corresponding risk for the decision maker’s risk-taking degree without reaching the Pareto optimality with the corresponding, which means the optimal inventory with random occurrence time will be higher than that with deterministic occurrence time. They will be decrease inventory to arrive the Pareto optimality, decreasing the cost but increasing the risk. If the probability of loss less than VaR with occurrence time is lower than that with deterministic occurrence time, the decision will be more risk-taking without reaching the Pareto states, which means the optimal inventory with random occurrence time will be lower than that with deterministic occurrence time. They will increase inventory to arrive the Pareto optimality, decreasing the risk but increasing the cost.

**Fig 8:**
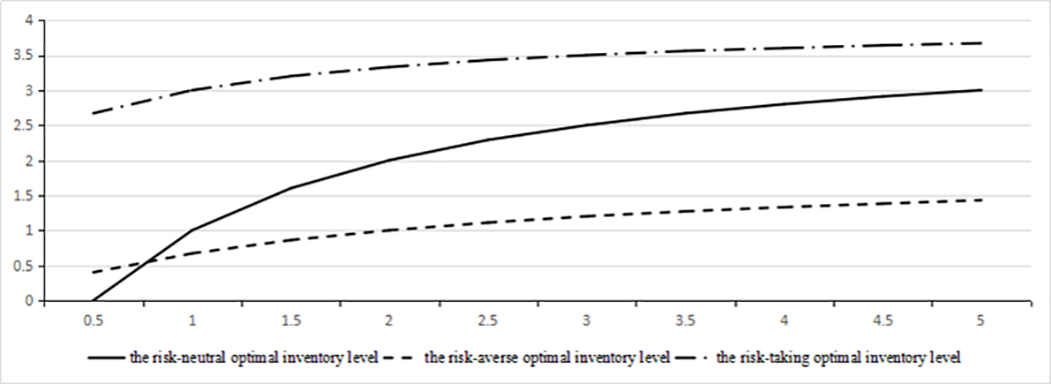
The sensitivity analysis of parameter for shortage cost s.

**Fig 9:**
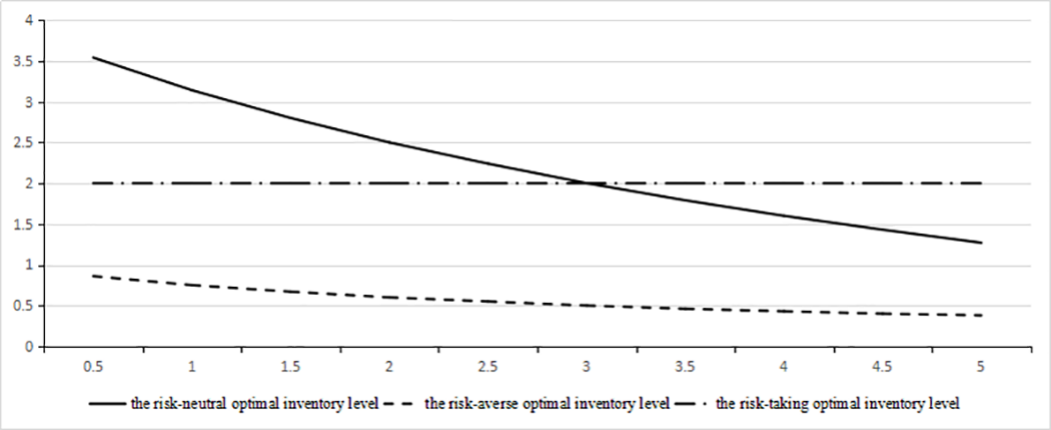
The sensitivity analysis of parameter for expiration cost e.

In addition, sensitivity analysis of parameters are computed, as shown in Figs 8 and 9. The parameters of shortage cost and expiration cost are the two important factors in our paper because of the two key losses from out-of-supply and out-of date. We find the risk-averse and risk-taking optimal inventory levels are more stable than that of risk-neutral model, which means solving risk-averse and risk-taking models may obtain the more reliable solutions, especially in violate situation.

## The extension of risk attitude related to probability of a disaster

Based on lots of cases, it can be inferred that the risk-averse degree increase with the increasing of the probability of a disaster. Based on literature [26], we always under- or overweight the actual probability. We assume the relationship between risk-averse degree and probability of a disaster is *η* = *Ap*^*β*^. *p* is the probability of a disaster during *T*, and *G*(*T*) = *p*.

$$1)\;\mathrm{Under}\;\mathrm{risk}\;\mathrm{neutral},\;I^{*}=F^{-1}(\min(1,{\left(\frac{(s+e)p-e}{(s+\theta e)p}\right)}^{+}))$$ (13)

$$2)\;\mathrm{Under}\;\mathrm{risk-aversion},\;\frac{(s+e)p-e}{s}<p^{\beta },I^{*}=F^{-1}({\left(1-\frac{1-p^{\beta }}{p}\right)}^{+})\frac{s}{s+e}$$ (14)

$$\frac{(s+e)p-e}{s}\geq p^{\beta },I^{*}=F^{-1}(\min(1,p^{\beta -1}))\frac{s}{s+\theta e}$$ (15)

$$3)\;\mathrm{Under}\;\mathrm{risk-taking}\;\frac{\left(s+e)(1-p\right)}{s}>1-p^{\beta },I^{*}=F^{-1}(\min(1,{\left(\frac{(s+e)p+sp^{\beta }-s-e}{(s+\theta e)p}\right)}^{+}))$$ (16)

$$\frac{\left(s+e)(1-p\right)}{s}\leq 1-p^{\beta },I^{*}=F^{-1}(\min(1,\frac{sp^{\beta \text{-}1}}{\left(s+\theta e\right)}))$$ (17)

Eqs (13) to (17), the optimal inventory level I* increase with increasing of probability *p*. When *p* become larger, the risk-averse degree is larger, increasing I* to balance risk. Obviously, from Eqs (14) to (17), the optimal inventory level I* decrease with increasing of *β*. When *β*≤1, the risk-averse degree is overweighed by the corresponding probability, and when *β*>1, the risk-averse degree is underweighted by the corresponding probability. If *β* is higher, the risk-averse degree is more underweighted than the actual probability, and the gap between I* with higher *β* and I* with lower *β* is larger. So intuitively, I* will also decrease with increasing of *β*.

To observe the effect of random occurrence time on decisions, we compare I* with $\bar{I}$ in classical newsboy model. Results are analyzed as follows.

1) Under risk neutral, $\bar{I}=F^{-1}(\frac{s}{s+e})$, $I^{*}=F^{-1}(\min(1,{\left(\frac{(s+e)p-e}{(s+\theta e)p}\right)}^{+}))$

There is ${\hat{p}}_{1}\in (\frac{e}{s+e},1)$, when $p\leq {\hat{p}}_{1}$, $\bar{I}\geq I^{*}$; when $p\geq {\hat{p}}_{1}$, $\bar{I}\leq I^{*}$.

2) Under risk-averse criterion, $\bar{I}=F^{-1}(\frac{(1\text{-}p^{\beta })s}{s+e})$

The existence of turning point $\hat{p}$ is dependent on parameters’ size. If $F^{-1}(0)\geq F^{\text{-}1}(1)\frac{s}{s+\theta e}$, there is no turning point $\hat{p}$ and always has $\bar{I}\geq I^{*}$. If $F^{-1}(0)\leq F^{\text{-}1}(1)\frac{s}{s+e}$, there is ${\hat{p}}_{2}\in (0,1)$, when $p\leq {\hat{p}}_{2}$, $\bar{I}\geq I^{*}$; when $p\geq {\hat{p}}_{2}$, $\bar{I}\leq I^{*}$.

3) Under risk-taking criterion

There is ${\hat{p}}_{3}\in (0,1)$, when $p\leq {\hat{p}}_{3}$, $\bar{I}\geq I^{*}$; when $p\geq {\hat{p}}_{3}$, $\bar{I}\leq I^{*}$.

These analyses show that the probability of a disaster during shelf life affects decisions and the decisions are the balance between risk and cost. We also find that the optimal inventory level under risk-averse criterion may not be higher than that with risk neutral and it depends on parameters’ value. However, the optimal inventory level under risk-taking criterion may not be lower than that with risk neutral criterion. In other words, the probability and the unit cost can make a risk-adverse decision-maker become a risk-taking decision-maker or make a risk-taking decision-maker become a risk-averse decision-maker. Because the optimal inventory level under risk-averse criterion may be lower than that with risk neutral criterion, which is the characteristic of risk-taking criterion. The values of $\tilde{p}$ are exist, for example: *β*≤1 (*β*>1), $\tilde{p}\to 0$

$$F^{-1}(\min(1,{\left(\frac{(s+e)\tilde{p}-e}{(s+\theta e)\tilde{p}}\right)}^{+}))\geq I^{*}=F^{-1}({\left(1-\frac{1-{\tilde{p}}^{\beta }}{\tilde{p}}\right)}^{+})\frac{s}{s+e}$$

$$\mathrm{Or}\;I^{*}=F^{-1}(\min(1,p^{\beta -1}))\frac{s}{s+\theta e}.$$

The results of numerical examples in Table 1 and Table 2 show the transformation between different risk attitudes, and prove our analysis.

**Table 1:** The optimal inventory level with different probability.

θ = 0.5,s = 3,e = 2,a = 2,b = 4,β = 2			
*p*	I* with risk neutral	I* with risk-adverse	I* with risk-taking
0.1	2	1.2	2
0.2	2	1.2	2
0.3	2	1.2	2
0.4	2	1.2	2
0.5	2.5	1.2	2
0.6	2.83	1.2	2
0.7	3.07	2.55	3.05
0.8	3.25	2.7	3.2
0.9	3.39	2.85	3.35
1	3.5	3	3.5

**Table 2:** The optimal inventory level with different probability.

θ = 0,s = 3,e = 2,a = 2,b = 4,β = 2			
*p*	I* with risk neutral	I* with risk-adverse	I* with risk-taking
0.1	2	2.2	2.2
0.2	2	2.4	2.4
0.3	2	2.6	2.6
0.4	2	2.8	2.8
0.5	2.7	3	3
0.6	3.1	3.2	3.2
0.7	3.43	3.4	3.4
0.8	3.66	3.6	3.6
0.9	3.85	3.8	3.8
1	4	4	4

## Conclusions and limitations

By the newsboy model, this paper considers the short shelf life and high requirement of medical emergency supplies for establishing emergency supplies reserves decision model of the emergency preparation phase. The model takes into account the uncertainty of demand and occurrence time in an aviation accident. Moreover, we compare stochastic with determinate occurrence time with risk neutral, risk-averse and risk-taking. According to the closed-form solutions and the results of numerical examples, some conclusion can be reached as follows.

1) The optimal inventory level emergency supplies is not only related to the material shelf life, also related to the distribution of random variables. In other words, we cannot obtain the optimal decisions if we overlook the demand uncertainty and occurrence time uncertainty.

2) The effect of stochastic occurrence time on the optimal decision under different risk criterion is different, that is to say, consideration of occurrence time uncertainty will make a big difference between decision-makers who has different risk attitudes. Thus, making sense of risk attitude of decision-maker is necessary.

3) The paper verifies again that decision under the rule of risk aversion is the result of balance between risks and costs, which can be interpreted by using the probability density function.

4) Experiment economists have researched the relationship between risk attitude and probability of events. We develop a model to measure risk attitude based on the probability of a disaster, and analyze the mechanism of how occurrence probability affects the emergency inventory management system.

The above conclusions may have a guiding function for inventory decision of airport emergency resources. However, there are a variety of ways to improve our limitations by future study. First, this emergency inventory system is studied by a static method, and it may be possible to extend multi-period newsboy problem [27–29]. Such an extension would track the results level of inventory dynamically. Second, only a single kind of resource is considered in this paper, but the fact is not like the case. The Emergency inventory system with many kinds of emergency resources need to be further studied [30]. Thirdly, the emergency or violate situation is full of uncertainty, such as capacity, lead-time, service rate, and so on. Thus, other stochastic factors should be researched in emergency inventory management system [31–32], which would be helpful for emergency decisions. Finally, this methodology also should be extended to measure risk attitude and losses of other disaster categories, like sudden infectious diseases. In addition, the shortage of empirical analysis is the biggest limitation for this paper, which should be included in the future to verify the model and give a serious guidance for airports’ emergency preparation.

## Supporting information

S1 Appendix (DOCX)
