# Supplementary material for: Medical resource inventory model for emergency preparation with uncertain demand and stochastic occurrence time under considering different risk preferences at the airport
Source: PLoS One. 2017 Sep 20;12(9):e0183472. doi: 10.1371/journal.pone.0183472 (PMC5606934; doi:10.1371/journal.pone.0183472)
Supplement: S1 Appendix — (DOCX) [file pone.0183472.s001.docx]

Appendix A

$I^{*}=F^{-1}(\min(1,(\frac{(s+e)G(T)-e}{(s+\theta e)G(T)})^{+})$, let$K=\frac{(s+e)G(T)-e}{(s+\theta e)G(T)}$, then:

$\partial K/\partial s=\frac{(\theta e-e)G^{2}(T)+eG(T)}{[(s+\theta e)G(T)]^{2}}$, $\partial K/\partial s>0$.

$\partial K/\partial e=\frac{(s-\theta s)G^{2}(T)-sG(T)}{[(s+\theta e)G(T)]^{2}}<0$, $\partial K/\partial\theta=\frac{-eG(T)}{[(s+\theta e)G(T)]^{2}}<0$.

Appendix B

$$\begin{matrix} L(I,v)=v+\frac{1}{1-\eta}\int_{0}^{T} \int_{I}^{+\infty} (s(x-I)-v)^{+}f(x)g(t)dxdt \\ +\frac{1}{1-\eta}\int_{0}^{T} \int_{0}^{I} (\theta e(I-x)-v)^{+}f(x)g(t)dxdt +\frac{1}{1-\eta}\int_{T}^{+\infty} \int_{0}^{+\infty} (eI-v)^{+}f(x)g(t)dxdt \end{matrix}$$

1) When$v\geq eI$, for 0≤*θ*≤1, we have$v\geq\theta eI$, and:

$$(s(x-I)-v)^{+}=\left\{\begin{matrix} sx-sI-v, x>(sI+v)/s \\ 0 x\leq(sI+v)/s \end{matrix}\right.$$

So, $L(I,v)=v+\frac{1}{1-\eta}\int_{0}^{T} \int_{I+v/s}^{+\infty} (s(x-I)-v)f(x)g(t)dxdt$

$\partial L(I,v)/\partial v=1-\frac{1}{1-\eta}\int_{0}^{T} \int_{I+v/s}^{+\infty} f(x)g(t)dxdt$ (B.1)

$$\partial^{2}L(I,v)/\partial v^{2}=\frac{G(T)}{1-\eta}\frac{1}{s}f(I+v/s)>0$$

$$\frac{\partial L(I,v)}{\partial v}|_{v=eI} =1-\frac{(1-F(I+\frac{e}{s}I))G(T)}{1-\eta}$$

Then: $\frac{\partial L(I,v)}{\partial v}|_{v=eI} :\left\{\begin{matrix} >0, (1-F(I+\frac{e}{s}I))G(T)<1-\eta(B.2) \\ \leq0, (1-F(I+\frac{e}{s}I))G(T)\geq1-\eta(B.3) \end{matrix}\right.$

2) When$\theta eI\leq v<eI$, we have:

$$(s(x-I)-v)^{+}=\left\{\begin{matrix} sx-sI-v, x>(sI+v)/s \\ 0 x\leq(sI+v)/s \end{matrix}\right.$$

$L(I,v)=v+\frac{1}{1-\eta}\int_{0}^{T} \int_{I+v/s}^{+\infty} (s(x-I)-v)f(x)g(t)dxdt+\frac{1}{1-\eta}\int_{T}^{+\infty} \int_{0}^{+\infty} (eI-v)f(x)g(t)dxdt$ $\partial L(I,v)/\partial v=1-\frac{1}{1-\eta}\int_{0}^{T} \int_{I+v/s}^{+\infty} f(x)g(t)dxdt -\frac{1}{1-\eta}\int_{T}^{+\infty} \int_{0}^{+\infty} f(x)g(t)dxdt$ (B.4)

$$\partial^{2}L(I,v)/\partial v^{2}=\frac{G(T)}{1-\eta}\frac{1}{s}f(I+v/s)>0$$

Notice that $\frac{\partial L(I,v)}{\partial v}|_{v=eI} =1-\frac{(1-F(I+\frac{e}{s}I))G(T)}{1-\eta}$

Because $\frac{\partial L(I,v)}{\partial v}|_{v=\theta eI} =1-\frac{1}{1-\eta}+\frac{1}{1-\eta}F(I+\frac{\theta e}{s}I)G(T)$

And notice that $\frac{\partial L(I,v)}{\partial v}|_{v=eI} :\left\{\begin{matrix} >0, (1-F(I+\frac{e}{s}I))G(T)<1-\eta(B.5) \\ \leq0, (1-F(I+\frac{e}{s}I))G(T)\geq1-\eta(B.6) \end{matrix}\right.$

Then $\frac{\partial L(I,v)}{\partial v}|_{v=\theta eI} :\left\{\begin{matrix} \leq0, \left\{\begin{matrix} equation(B.6) is satisified \text{（}B.7\text{）} \\ equation(B.5) is satisified and F(I+\frac{\theta e}{s}I)G(T)\leq\eta(B.8) \end{matrix}\right. \\ >0, equation(B.5) is satisified and F(I+\frac{\theta e}{s}I)G(T)>\eta(B.9) \end{matrix}\right.$

3) When$v<\theta eI$, for 0≤θ≤1, we have$v<eI$, and:

$$(s(x-I)-v)^{+}=\left\{\begin{matrix} sx-sI-v, x>(sI+v)/s \\ 0 x\leq(sI+v)/s \end{matrix}\right.$$

$$(\theta e(I-x)-v)^{+}=\left\{\begin{matrix} \theta eI-\theta ex-v, x<(\theta eI-v)/\theta e \\ 0 x\geq(\theta eI-v)/\theta e \end{matrix}\right.$$

$$\begin{matrix} L(I,v)=v+\frac{1}{1-\eta}\int_{0}^{T} \int_{I+v/s}^{+\infty} (s(x-I)-v)f(x)g(t)dxdt \\ +\frac{1}{1-\eta}\int_{0}^{T} \int_{0}^{I-v/\theta e} (\theta e(I-x)-v)f(x)g(t)dxdt +\frac{1}{1-\eta}\int_{T}^{+\infty} \int_{0}^{+\infty} (eI-v)f(x)g(t)dxdt \end{matrix}$$

$\begin{matrix} \partial L(I,v)/\partial v=1-\frac{1}{1-\eta}\int_{0}^{T} \int_{I+v/s}^{+\infty} f(x)g(t)dxdt-\frac{1}{1-\eta}\int_{0}^{T} \int_{0}^{I-v/\theta e} f(x)g(t)dxdt \\ -\frac{1}{1-\eta}\int_{T}^{+\infty} \int_{0}^{+\infty} f(x)g(t)dxdt \end{matrix}$ (B.10)

$$\partial^{2}L(I,v)/\partial v^{2}=\frac{G(T)}{1-\eta}[\frac{1}{s}f(I+v/s)+\frac{1}{\theta e}f(I-v/\theta e)]>0$$

Notice that $\frac{\partial L(I,v)}{\partial v}|_{v=\theta eI} =1-\frac{1}{1-\eta}+\frac{1}{1-\eta}F(I+\frac{\theta e}{s}I)G(T)$

Then $\frac{\partial L(I,v)}{\partial v}|_{v=\theta eI} :\left\{\begin{matrix} \leq0, \left\{\begin{matrix} equation(B.7) is satisified \text{（}B.11\text{）} \\ equation(B.8) is satisified (B.12) \end{matrix}\right. \\ >0, equation(B.9) is satisified (B.13) \end{matrix}\right.$

Note $\bar{v}(I)$ is the optimal solution for some fixed *I*, then from Figure 8, we can see that equation (B.11)、(B.12) and (B.13) cannot get the optimal solution at the same time.

v

L(I.v)

θeI

eI

v

L(I.v)

θeI

eI

v

L(I.v)

θeI

eI

(a) the case of equation(B.11) (b) the case of equation(B.12) (c) the case of equation(B.13)

Figure 8. The optimal solutions under different cases

1) When equation (B.11) is satisfied, then$\bar{v}(I)\geq eI$. we can have:

If equation (B.1) equal to zero where *v*=$\bar{v}(I)$, then:

So, $I^{*}=F^{-1}((1-\frac{1-\eta}{G(T)})^{+})\frac{s}{s+e}$,$\bar{v}(I)=(F^{-1}((1-\frac{1-\eta}{G(T)})^{+})-I)s$ (B.14)

2) When equation (B.12) is satisfied, then$\theta eI\leq\bar{v}(I)<eI$.

If equation (B.4) equal to zero where *v*=$\bar{v}(I)$, then:

(B.15)

Let equation (B.15) equal to zero, then$\bar{v}^{*}(I)=(F^{-1}(\frac{(s+e)G(T)-e}{sG(T)})-I)s$

And $dL(I,\bar{v}^{*}(I))/dI=-s(1-\frac{1}{1-\eta}(1-\frac{(s+e)G(T)-e}{s}))$ (B.16)

ⅰ. When equation (B.16) is positive,$\frac{(s+e)G(T)-e}{s}<\eta$, then:

$\bar{v}(I)=eI$,$I^{*}=F^{-1}((1-\frac{1-\eta}{G(T)})^{+})\frac{s}{s+e}$ (B.17)

ⅱ. When equation (B.16) is negative,$\frac{(s+e)G(T)-e}{s}>\eta$, then:

$I^{*}=F^{-1}(\min(1,\frac{\eta}{G(T)}))\frac{s}{s+\theta e}$,$\bar{v}(I)=(F^{-1}(\min(1,\frac{\eta}{G(T)}))-I)s$ (B.18)

ⅲ. When equation (B.16) is equal to zero,$\frac{(s+e)G(T)-e}{s}=\eta$, then the optimal value *I** can be arbitrary value during$(F^{-1}(\min(1,\frac{\eta}{G(T)}))\frac{s}{s+e},F^{-1}(\min(1,\frac{\eta}{G(T)}))\frac{s}{s+\theta e}]$. (B.19)

3) When equation (B.13) is satisfied, then$\bar{v}(I)<\theta eI$

If equation (B.10) equal to zero where *v*=$\bar{v}(I)$, then:

(B.20)

Let equation (B.20) equal to zero, and then$\bar{v}^{*}(I)$ satisfy the follow relationship:

According to the derivation rule of implicit function, we have$d\bar{v}^{*}(I)/dI\leq0$.

(B.21)

Let equation (B.21) equal to zero, then:

$I^{*}=\frac{sF^{-1}(\frac{(s+e)G(T)-e+\theta e\eta}{(s+\theta e)G(T)})+\theta eF^{-1}(\frac{(s+e)G(T)-e-s\eta}{(s+\theta e)G(T)})}{s+\theta e}$ (B.22)

$L(I,\bar{v}(I))$ is the convex function of *I*, so *I** is the unique optimal solution.

Since$I>F^{-1}(\min(1,\frac{\eta}{G(T)}))\frac{s}{s+\theta e}$, so the optimal solution should satisfy the follow relationship:

$$\begin{matrix} I^{*}=\frac{sF^{-1}(\min(1,(\frac{(s+e)G(T)-e+\theta e\eta}{(s+\theta e)G(T)})^{+}))+\theta eF^{-1}(\min(1,(\frac{(s+e)G(T)-e-s\eta}{(s+\theta e)G(T)})^{+}))}{s+\theta e} \\ \geq F^{-1}(\min(1,\frac{\eta}{G(T)}))\frac{s}{s+\theta e} \end{matrix}$$

$$\begin{matrix} F^{-1}(\min(1,\frac{\eta}{G(T)})) \\ \leq F^{-1}(\min(1,(\frac{(s+e)G(T)-e+\theta e\eta}{(s+\theta e)G(T)})^{+}))+\frac{\theta e}{s}\theta eF^{-1}(\min(1,(\frac{(s+e)G(T)-e-s\eta}{(s+\theta e)G(T)})^{+})) \end{matrix}$$

Otherwise$I^{*}=F^{-1}(\min(1,\frac{\eta}{G(T)}))\frac{s}{s+\theta e}$

$$I^{*}=\frac{sF^{-1}(\min(1,(\frac{(s+e)G(T)-e+\theta e\eta}{(s+\theta e)G(T)})^{+}))+\theta eF^{-1}(\min(1,(\frac{(s+e)G(T)-e-s\eta}{(s+\theta e)G(T)})^{+}))}{s+\theta e}$$

$\bar{v}^{*}(I^{*})=\frac{s\theta e}{s+\theta e}(F^{-1}(\min(1,(\frac{(s+e)G(T)-e+\theta e\eta}{(s+\theta e)G(T)})^{+}))+F^{-1}(\min(1,(\frac{(s+e)G(T)-e-s\eta}{(s+\theta e)G(T)})^{+})))$ $\bar{v}^{*}(I^{*})\geq\theta eI*$, this is contrary with$\bar{v}(I)<\theta eI$, so we should give up this optimal solution.

$\bar{v}(I)=\theta eI$,$I^{*}=F^{-1}(\min(1,\frac{\eta}{G(T)}))\frac{s}{s+\theta e}$ (B.23)

Above all$I^{*}=F^{-1}((1-\frac{1-\eta}{G(T)})^{+})\frac{s}{s+e}$、$I^{*}=F^{-1}(\min(1,\frac{\eta}{G(T)}))\frac{s}{s+\theta e}$ or arbitrary value during $(F^{-1}(\min(1,\frac{\eta}{G(T)}))\frac{s}{s+e},F^{-1}(\min(1,\frac{\eta}{G(T)}))\frac{s}{s+\theta e}]$.

Appendix C

Let$K_{1}=\bar{I}-I^{*}=F^{-1}(\frac{(1-\eta)s}{s+e})-F^{-1}((1-\frac{1-\eta}{G(T)})^{+})\frac{s}{s+e}$, then:

$\partial K_{1}/\partial\eta<0$, since$\frac{(s+e)G(T)-e}{s}<\eta$and$G(T)\geq1-\eta$, when$G(T)\leq\frac{s+e}{2s+e}$, we have:

$\eta\geq1-G(T)$, and$K_{1}|_{\eta=1-G(T)}>0,K_{1}|_{\eta=1}<0$.

So there is$\hat{\eta}_{1}\in(1-G(T),1)$, satisfying. Then when$\eta\leq\hat{\eta}_{1}$, $\bar{I}\geq I^{*}$; when $\eta\geq\hat{\eta}_{1}$, $\bar{I}\leq I^{*}$.

When$G(T)\geq\frac{s+e}{2s+e}$, we have$\eta\geq\frac{(s+e)G(T)-e}{s}$, $K_{1}|_{\eta=\frac{(s+e)G(T)-e}{s}}>0,K_{1}|_{\eta=1}<0$. So there is$\hat{\eta}_{1}\in(\frac{(s+e)G(T)-e}{s},1)$, satisfying. Then when$\eta\leq\hat{\eta}_{1}$, $\bar{I}\geq I^{*}$; when$\eta\geq\hat{\eta}_{1}$, $\bar{I}\leq I^{*}$.

Let$K_{2}=\bar{I}-I^{*}=F^{-1}(\frac{(1-\eta)s}{s+e})-F^{-1}(\min(1,\frac{\eta}{G(T)}))\frac{s}{s+\theta e}$, then:

$\partial K_{2}/\partial\eta<0$, since$\frac{(s+e)G(T)-e}{s}>\eta$ and$G(T)\geq\eta$, that is, $\eta<\frac{(s+e)G(T)-e}{s}$. We have$K_{2}|_{\eta=0}>0,K_{2}|_{\eta=\frac{(s+e)G(T)-e}{s}}>0$. So there is not $\hat{\eta}_{2}\in(0,\frac{(s+e)G(T)-e}{s})$ satisfying , and for any value satisfying$\eta<\frac{(s+e)G(T)-e}{s}$, we have$\bar{I}\geq I^{*}$.

When$G(T)\leq\frac{s+e}{2s+e}$ and$\frac{(s+e)G(T)-e}{s}<\eta$, there is$\hat{\eta}_{1}\in(1-G(T),1)$, when$\eta\leq\hat{\eta}_{1}$, $\bar{I}\geq I^{*}$; when$\eta\geq\hat{\eta}_{1}$, $\bar{I}\leq I^{*}$.

When$G(T)\geq\frac{s+e}{2s+e}$ and$\frac{(s+e)G(T)-e}{s}<\eta$, there is $\hat{\eta}_{1}\in(\frac{(s+e)G(T)-e}{s},1)$，when $\eta\leq\hat{\eta}_{1}$, $\bar{I}\geq I^{*}$; when$\eta\geq\hat{\eta}_{1}$, $\bar{I}\leq I^{*}$.

When$\eta<\frac{(s+e)G(T)-e}{s}$, $\bar{I}\geq I^{*}$.

Appendix D

When$0\leq\alpha\leq\frac{(s+e)(1-G(T))}{s}$

Let$N_{1}=\bar{I}-I^{*}=F^{-1}(\frac{\alpha s}{s+e})-F^{-1}(\min(1,(\frac{(s+e)G(T)-\alpha s-e}{(s+\theta e)G(T)})^{+}))$, then:

$\partial N_{1}/\partial\alpha>0$,$N_{1}|_{\alpha=0}<0,$

$N_{1}|_{\alpha=\frac{(s+e)(1-G(T))}{s}}=F^{-1}(1-G(T))-F^{-1}(\frac{2(s+e)G(T)-(s+2e)}{(s+\theta e)G(T)})$.

Let$M_{1}=(s+\theta e)G(T)(1-G(T))-(2(s+e)G(T)-(s+2e))$

$\partial M_{1}/\partial G(T)<0$,$M_{1}|_{G(T)=0}>0$, $M_{1}|_{G(T)=1}<0$, so there is$G_{1}(T)\in(0,1)$,$M_{1}|_{G_{1}(T)}=0$.

When$G(T)\geq G_{1}(T)$,$N_{1}|_{\alpha=\frac{(s+e)(1-G(T))}{s}}\leq0$, we always have$\bar{I}\leq I^{*}$.

When$G(T)\leq G_{1}(T)$,$N_{1}|_{\alpha=\frac{(s+e)(1-G(T))}{s}}\geq0$, there is$\alpha_{1}\in(0,\frac{(s+e)(1-G(T))}{s})$,$N_{1}|_{\alpha_{1}}=0$. And when$0\leq\alpha\leq\alpha_{1}$, we have$\bar{I}\leq I^{*}$. When$\alpha_{1}\leq\alpha\leq\frac{(s+e)(1-G(T))}{s}$, we have$\bar{I}\geq I^{*}$.

When$\frac{(s+e)(1-G(T))}{s}\leq\alpha\leq1$

Let$N_{2}=\bar{I}-I^{*}=F^{-1}(\frac{\alpha s}{s+e})-F^{-1}(\min(1,\frac{(1-\alpha)s}{(s+\theta e)G(T)}))$

$\partial N_{2}/\partial\alpha>0$,$N_{2}|_{\alpha=1}>0,$

$N_{2}|_{\alpha=\frac{(s+e)(1-G(T))}{s}}=F^{-1}(1-G(T))-F^{-1}(\frac{(s+e)G(T)-e}{(s+\theta e)G(T)})$.

Let$M_{2}=(s+\theta e)G(T)(1-G(T))-((s+e)G(T)-e)$

$\partial M_{2}/\partial G(T)<0$,$M_{2}|_{G(T)=0}>0$, $M_{2}|_{G(T)=1}<0$, so there is$G_{2}(T)\in(0,1)$,$M_{2}|_{G_{2}(T)}=0$.

When$G(T)\leq G_{2}(T)$, $N_{2}|_{\alpha=\frac{(s+e)(1-G(T))}{s}}\geq0$we always have$\bar{I}\geq I^{*}$.

When$G(T)\geq G_{2}(T)$,$N_{2}|_{\alpha=\frac{(s+e)(1-G(T))}{s}}\leq0$, there is$\alpha_{2}\in(\frac{(s+e)(1-G(T))}{s},1)$,$N_{1}|_{\alpha_{1}}=0$. And when$\alpha_{2}\leq\alpha\leq1$, we have$\bar{I}\geq I^{*}$. When$\frac{(s+e)(1-G(T))}{s}\leq\alpha\leq\alpha_{2}$, we have$\bar{I}\leq I^{*}$.
